# The anticancer drug mithramycin A sensitises tumour cells to apoptosis induced by tumour necrosis factor (TNF)

**DOI:** 10.1038/sj.bjc.6601824

**Published:** 2004-04-20

**Authors:** V Duverger, A-M Murphy, D Sheehan, K England, T G Cotter, I Hayes, F J Murphy

**Affiliations:** 1 EiRx Therapeutics Ltd, 2800 Cork Airport Business Park, Kinsale Road, Cork, Ireland; 2 Department of Biochemistry, Biosciences Institute, University College, Cork, Ireland

**Keywords:** mithramycin A, GM-CSF, TNF, anti-Fas, apoptosis, bcl-2

## Abstract

In this report we show that mithramycin considerably increases the direct cytotoxic effect of tumour necrosis factor (TNF) on tumour cells *in vitro*. Sensitisation to TNF-induced apoptosis was prevented by the broad caspase inhibitor zVAD-fmk, whereas overexpression of Bcl-2 had no effect. Mithramycin also potentiated cell death induced by Fas agonistic antibodies. In contrast, mithramycin reduced the percentage of cells undergoing apoptosis due to factor withdrawal. TNF-induced activation of NF-kappaB (NF-*κ*B)-dependent gene expression was not modulated by mithramycin treatment. Concomitantly with the increased sensitivity, the protein level of the short-spliced cFLIP variant was downregulated. These results indicate that mithramycin enhances TNF-induced cell death in an NF-*κ*B-independent manner, and suggest that the Fas-associated death domain protein plays a crucial role in the TNF-sensitising effect of mithramycin.

Mithramycin A (trade name Plicamycin) is an anticancer drug used to treat several types of cancer including chronic myeloid leukaemia and acute myeloid leukaemia (Koller and Miller, 1986; Queisser *et al*, 1988). Its mechanism of action involves a reversible interaction with double-stranded DNA with GC base specificity. It is believed to act, in part, by selectively inhibiting transcription of genes that have GC-rich promoter sequences (Miller *et al*, 1987). In this regard, mithramycin has been shown to prevent resistance to chemotherapeutic agents, by a number of mechanisms including downregulation of proteins, such as multidrug resistance gene 1 (Tagashira *et al*, 2000). Since most chemotherapeutic drugs are known to activate the process of apoptosis, dysregulation of this process can affect treatment sensitivity. Understanding the mechanism of action of mithramycin in combination with various apoptotic stimuli could lead to strategies resulting in improved therapeutic benefits.

Cells are responsive to a multitude of signals that they encounter in their extracellular environment. One such signal with widespread pleiotropic actions is the cytokine tumour necrosis factor-alpha (TNF). Differential responses to this cytokine are thought to be due to different intracellular signalling pathways (Leong and Karsan, 2000). One major signalling pathway leads to the induction of apoptosis through the activation of caspase-8 or -10 via TNF receptor-associated death domain (TRADD) and Fas-associated death domain (FADD) (Hsu *et al*, 1996). Activated caspases then cleave downstream substrates that initiate the execution of apoptosis, such as caspase-3, or amplify the programmed cell death signal, through cleavage of BID and activation of the mitochondrial apoptotic pathway. Apoptosis initiated by Fas triggering follows a similar pathway, but in this case FADD is directly recruited to the Fas receptor without the involvement of TRADD (Chinnaiyan *et al*, 1996). To prevent inappropriate apoptosis, the death receptor signalling is controlled at different checkpoints: namely at the receptor level by modulation of the receptor, or soluble ligands; during signal transduction by FLIP and Bcl-2 proteins, or during the effector phase by inhibitors of apoptosis (IAPs). Another major signalling pathway leads to the activation of the transcription factor nuclear factor-kappaB (NF-*κ*B). Nuclear factor-kappaB is believed to be activated via TRADD and TNF receptor-associated factor-2(TRAF2), and/or receptor interacting protein (RIP) (Kelliher *et al*, 1998). Activation of NF-*κ*B induces protective mechanisms by stimulation of the expression of regulator proteins with potential antiapoptotic activity, such as the IAP proteins (Beg and Baltimore, 1996; Van Antwerp *et al*, 1996).

Tumour necrosis factor receptors are expressed on a variety of different cell types, yet many TNF-receptor-expressing cells are resistant to TNF-induced apoptosis. The latter and the proinflammatory effects of TNF that lead to several toxic side effects *in vivo*, have limited the use of TNF as an antitumour agent (Kettelhut *et al*, 1987). However, combination treatments that allow the use of lower nontoxic doses and/or prevent cell resistance might still offer opportunities for TNF in the treatment of cancer (Wajant *et al*, 2000; Schotte *et al*, 2001). In view of the reported use of mithramycin A in preventing chemotherapy resistance, we investigated the effect of mithramycin in TNF-induced cell death.

## MATERIALS AND METHODS

### Cell lines and reagents

All human cell lines were obtained from the ATCC. TF-1, a human GM-CSF-dependent erythroleukaemic cell line (Kitamura *et al*, 1989), was maintained in logarithmic growth at 2 × 10^5^ cells ml^−1^ in complete RPMI 1640 medium containing 10% foetal calf serum (FCS) and 10% hGM-CSF-conditioned medium (complete medium). hGM-CSF-conditioned medium was obtained from stably transduced human embryonic kidney 293 cells with a pLHCX plasmid (Clontech, Oxford, UK) encoding the human GM-CSF protein. The HL60 promyelocytic leukaemia and U937 human histiocytic lymphoma cell lines were maintained in RPMI 1640 medium containing 10% FCS. The GM-CSF producing HEK-293 cells were maintained in Dulbecco's modified Eagle's medium (DMEM) supplemented with 10% FCS. The caspase inhibitor zVAD-fmk, the transcription inhibitor actinomycin D and the antimitotic agent aphidicolin were supplied by Sigma, Dublin, Irl. Recombinant human TNF was purchased from R&D systems, Abington, UK. Anti-Fas antibodies (agonistic antibodies; clone CH-11) and anti-FLIP *γ*/*δ* antibodies were obtained from MBL, MA, USA and Calbiochem, Nottingham, UK respectively. Mithramycin A was purchased from Tocris, Bristol, UK.

### Cell treatments

For GM-CSF withdrawal experiments, TF-1 cells were washed twice in Hank's buffered saline solution, then resuspended in RPMI 1640 medium containing 10% FCS in the presence or absence of 75 nM mithramycin. For combination experiments with TNF or anti-Fas, cells were maintained in complete medium and treated with mithramycin simultaneously to the different drugs.

### Quantification of apoptosis

Cell cycle status and quantification of DNA fragmentation was performed by propidium iodide (PI) staining according to Darzynkiewicz (Darzynkiewicz *et al*, 2001). DNA content was determined using a FACSCalibur (Becton Dickinson, Oxford, UK) flow cytometer. Data were analysed by Cellquest software. Quantification of apoptosis by phosphatidylserine exposure was assessed by annexin V staining using the Alexa Flour 488 kit (Molecular Probes, Rijnsburgerweg, The Netherlands), according to the manufacturer's instruction.

### Generation of cells stably expressing an NF-*κ*B reporter plasmid

TF-1 cells were stably transfected with the pNF-*κ*B-hrGFP plasmid (Stratagene, Amsterdam, The Netherlands) expressing a hrGFP gene controlled by a synthetic promoter that contains five binding sites for NF-*κ*B. A measure of 5 *μ*g of reporter plasmid was transfected with lipofectin (Gubina *et al*, 1998).

### Western blot analysis

Proteins were extracted in RIPA buffer (50 mM Tris, 1% NP40, 150 mM NaCl, 1 mM EGTA) with complete protease inhibitor mix from Roche, East Sussex, UK. In total, 30 *μ*g of protein were separated by sodium dodecyl sulphate–polyacrylamide gel electrophoresis (12% SDS–PAGE), transferred to a nitrocellulose membrane and immunoblotted with the indicated antibody. Immunoreactive proteins were detected by enhanced chemiluminescence (ECL, Amersham, Bucks, UK).

### Statistical analysis

All results were expressed as the mean±s.d. of three independent experiments performed in duplicate. These data were analysed using Student's *t-*test for paired comparisons. Statistical significance was determined as *P*<0.05.

## RESULTS

### Co-treatment with mithramycin increases the cytotoxicity effect of TNF in tumour cells

**Figure 1:**
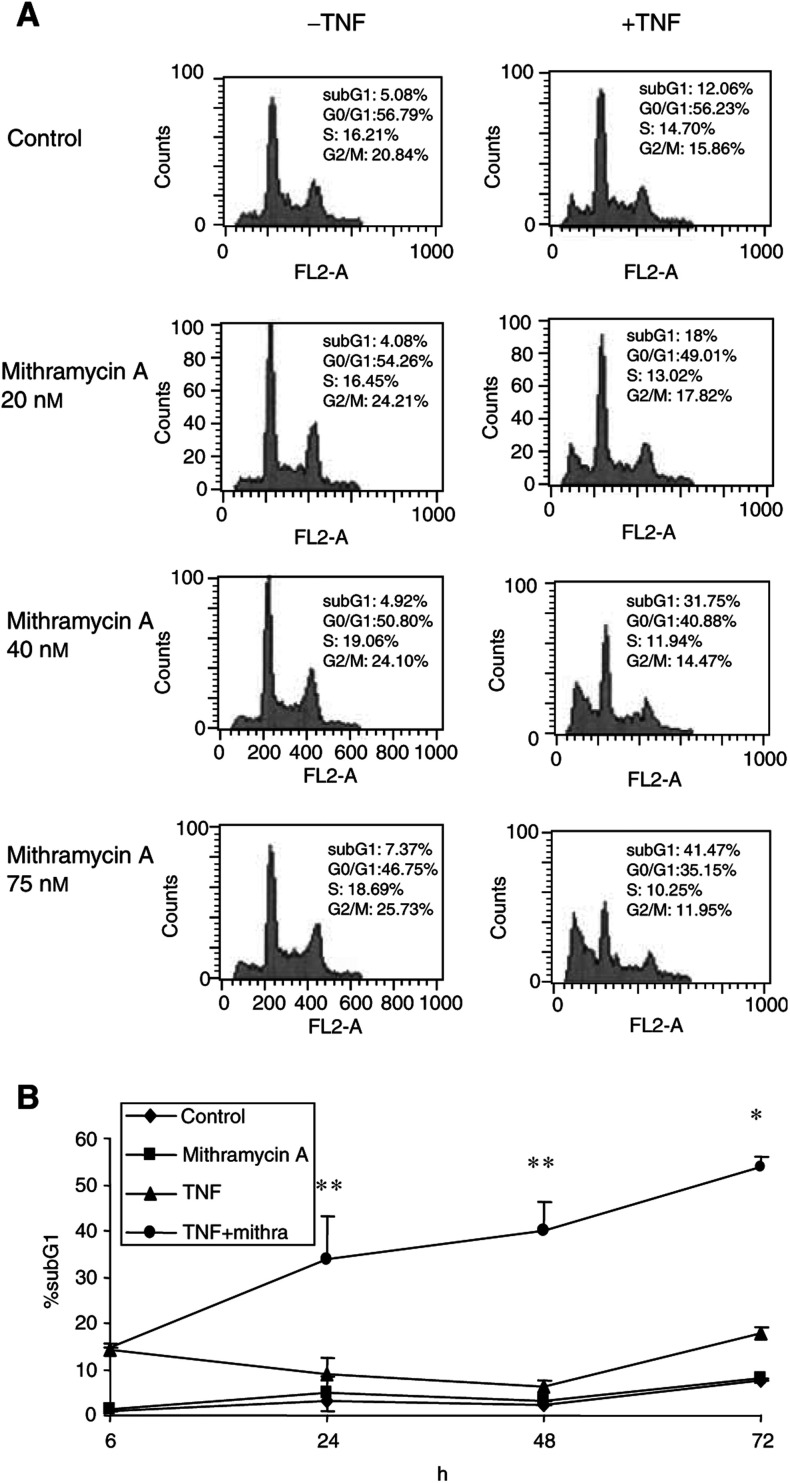
Mithramycin increased TNF-induced cell death in TF-1 cells. (**A**) TF-1 cells were treated with 20 ng ml^−1^ TNF-alpha (TNF) in the presence or absence of increasing concentrations of mithramycin. After 48 h of incubation, DNA profiles were established by flow cytometry following PI staining. (**B**) TF-1 cells were treated for the indicated periods with 20 ng ml^−1^ TNF in the presence or absence of 75 nM mithramycin. Percentage of subG1 cells was determined by PI staining of ethanol-fixed cells and analysed by flow cytometry. ^*^, *P*<0.05; ^**^, *P*<0.01 of three independent experiments compared to TNF-treated cells.

Since the TF-1 erythroleukaemia cell line is relatively resistant to apoptosis induced by TNF, we first investigated whether sublethal concentrations of mithramycin would affect cells sensitivity to TNF-mediated apoptosis. Cell death, that is the percentage of cells with fragmented DNA in subG1, was quantified by flow cytometry following PI staining. As shown in Figure 1A

, there was little effect on DNA fragmentation and cell cycle distribution after 48 h of exposure to either agent alone. However, in the presence of both mithramycin and TNF, TF-1 cells underwent significant cell death. Mithramycin-mediated sensitisation was dose dependent and was detected after 24 h incubation with TNF (Figure 1B).

**Figure 2:**
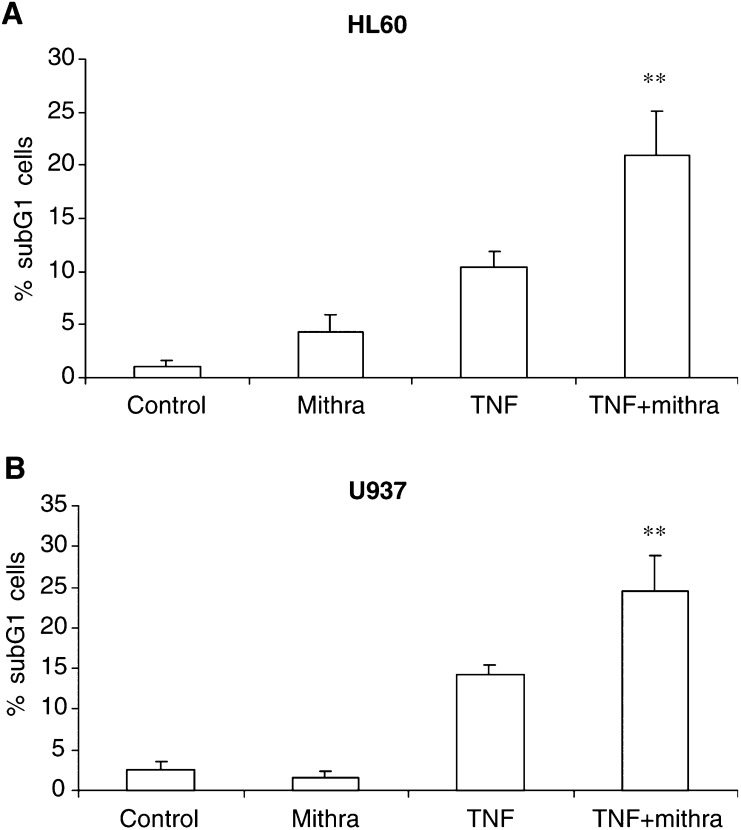
Mithramycin increased TNF-induced cell death in HL60 and U937 cells. HL60 (**A**) and U937 (**B**) cells were treated with 20 ng ml^−1^ TNF in the presence or absence of 75 nM mithramycin. Percentage of subG1 cells was determined by PI staining and analysed by flow cytometry. ^**^, *P*<0.01 of three independent experiments compared to TNF-treated cells.

To study whether the observed sensitisation was specific to TF-1 cells, we tested the TNF sensitivity of two additional cell lines: HL60 promyelocytic leukaemia and the U937 human histiocytic lymphoma cell lines (Figure 2

). Both cell lines displayed modest sensitivity to TNF. However, when the cells were cotreated with mithramycin, their sensitivity towards TNF was doubled as quantified by flow cytometry.

To further elucidate the sensitisation effects of mithramycin, we focused our studies on the erythroleukaemic cell line TF-1.

### Sensitisation to TNF-induced apoptosis by mithramycin is caspase dependent but is not prevented by Bcl-2 overexpression

**Figure 3:**
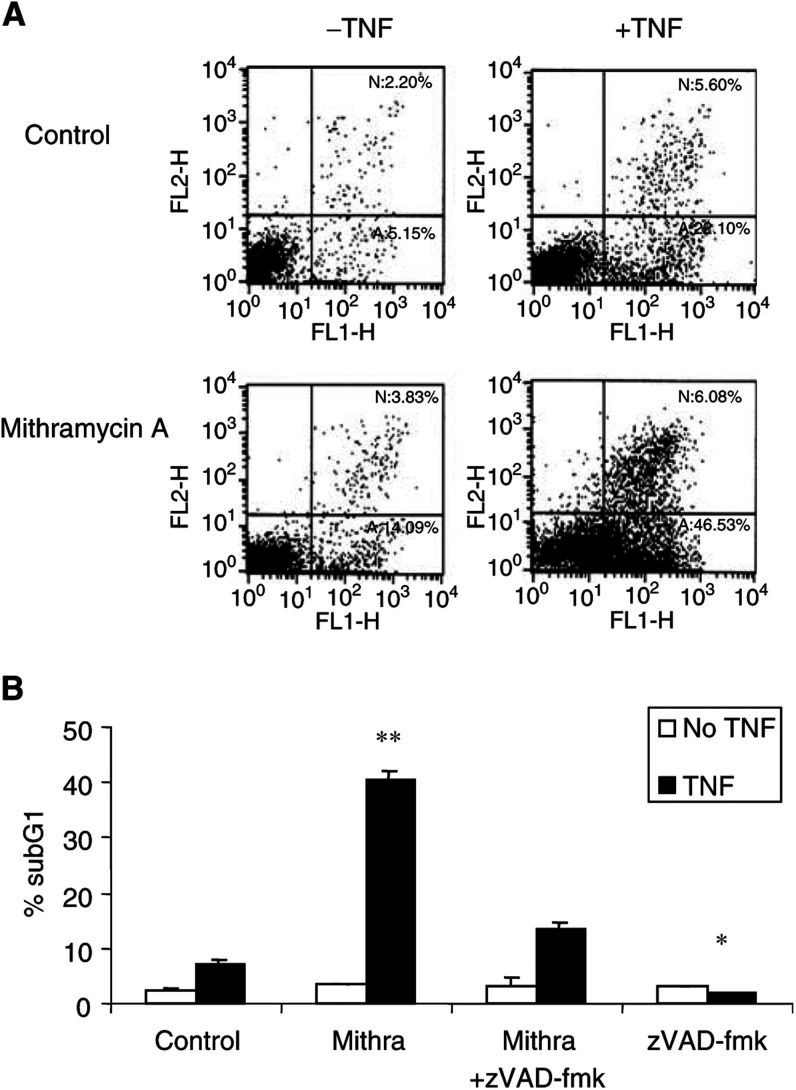
Sensitisation to TNF-induced apoptosis by mithramycin is caspase dependent. TF-1 cells were treated with 20 ng ml^−1^ TNF in the presence or absence of 75 nM mithramycin. (**A**) After 24 h of incubation, cells were stained with annexin V and PI. Necrosis (N) and apoptosis (A) were quantified by flow cytometry. The annexin V-positive cells are the cells undergoing apoptosis and are represented in the lower right quadrant. Similar results were obtained from two more independent experiments. (**B**) Effect of 50 *μ*M caspase inhibitor zVAD-fmk. Percentage of subG1 cells was determined by PI staining and analysed by flow cytometry. ^*^, *P*<0.05; ^**^, *P*<0.01 of three independent experiments compared to TNF-treated cells.

To confirm that the cell death induced by mithramycin in TNF-treated cells was due to apoptosis, TF-1 cells were simultaneously stained with PI and annexin V, and analysed by flow cytometry (Figure 3A

). Following TNF treatment, 22.10% of cells stained positive with annexin V and negative with PI, indicating that they were undergoing apoptosis. At this time point, an additional 5.60% of the cells stained positive for both annexin V and PI, representing cells undergoing late apoptosis/secondary necrosis. Cotreatment with mithramycin increased the percentage of cells stained positive for annexin V and negative for PI to 46.33%, whereas cells positive for both PI and annexin remain relatively unchanged (6.08%).

To evaluate the role of caspases in mithramycin-mediated sensitisation to TNF, we treated TF-1 cells with TNF and/or mithramycin in the presence of the caspase inhibitor, z-VAD-fmk. Treatment of cells with 50 *μ*M z-VAD-fmk prevented mithramycin/TNF-induced DNA fragmentation (Figure 3B).

**Figure 4:**
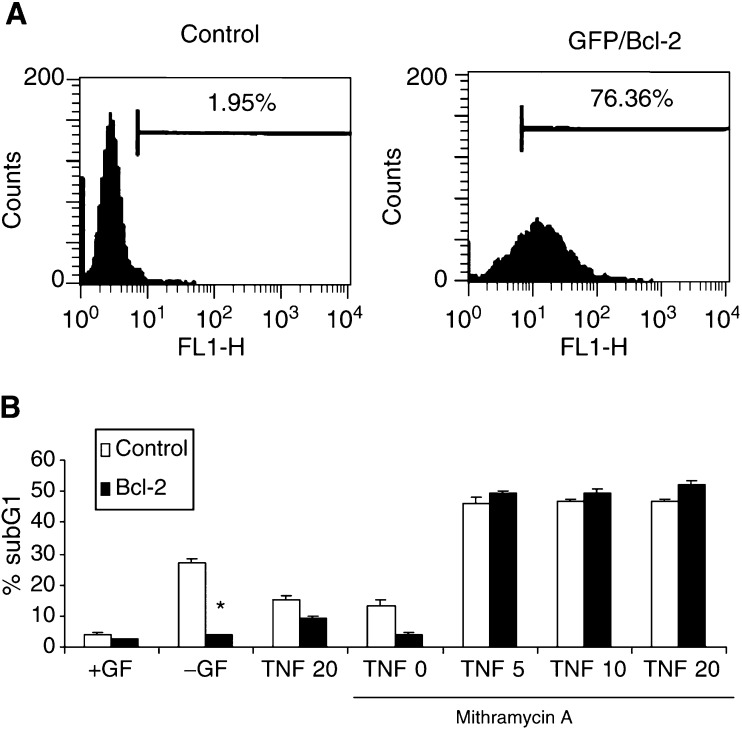
Sensitisation to TNF-induced apoptosis by mithramycin is not prevented by Bcl-2 overexpression. (**A**) Analysis of GFP expression in TF-1 cells. Mock-transfected (control) TF-1 cells and cells stably transfected with GFP/bcl-2 expression vector were analysed by flow cytometry. GFP/bcl-2-transfected cells were identified by their GFP fluorescence. (**B**) Mock-transfected (control) TF-1 cells and cells stably transfected with GFP/bcl-2 (bcl-2) were depleted of GM-CSF or treated with increasing concentrations of TNF in the presence or absence or 75 nM mithramycin. After 24 h of incubation, percentage of subG1 cells were determined by PI staining and analysed by flow cytometry. Data are representative of three independent experiments performed in duplicate. ^*^, *P*<0.05 compared to GM-CSF-depleted mock-transfected cells.

Next, we examined whether mithramycin-mediated sensitisation was modulated by overexpression of Bcl-2. Bcl-2 is believed to prevent caspase activation by blocking the mitochondrial pathway (Yang *et al*, 1997). A vector containing the green fluorescent protein (GFP) gene and the antiapoptotic bcl-2 gene together with an internal ribosome entry site (IRES) sequence was used, allowing us to analyse the percentage of cells stably expressing GFP/Bcl-2. As represented in Figure 4A

, more than 76% of GFP/Bcl-2-transfected cells expressed GFP, and this percentage was not modulated in the presence of mithramycin, demonstrating that mithramycin was not affecting expression of the bcl-2 gene. To further establish whether expressed Bcl-2 was functionally active, cells were depleted with GM-CSF. The TF-1 cell line has been reported to undergo apoptosis upon growth factor deprivation (Kolonics *et al*, 2001), which can be suppressed by overexpression of Bcl-2 (Kitamura *et al*, 1989; Alvarado-Moreno *et al*, 2000). Upon GM-CSF withdrawal (Figure 4B), 30% of the control cell population underwent apoptosis as determined by the number of cells with fragmented DNA (subG1), whereas in bcl-2-transfected cells DNA fragmentation was inhibited completely. Therefore, the bcl-2-transfected cells, which were recognised by their GFP fluorescence, overexpressed a functional Bcl-2 protein. TF-1 cells overexpressing Bcl-2 were treated with increasing concentrations of TNF in the presence or absence of mithramycin for 24 h, and then assessed for DNA fragmentation by flow cytometry. As shown in Figure 4B, Bcl-2 overexpression did not prevent mithramycin-mediated sensitisation to TNF. In agreement with these results, no loss of the mitochondrial membrane potential was observed following 24 h treatment with TNF and mithramycin (data not shown).

### Cotreatment with mithramycin increases the cytotoxic effect of anti-Fas antibodies in TF-1 cells

**Figure 5:**
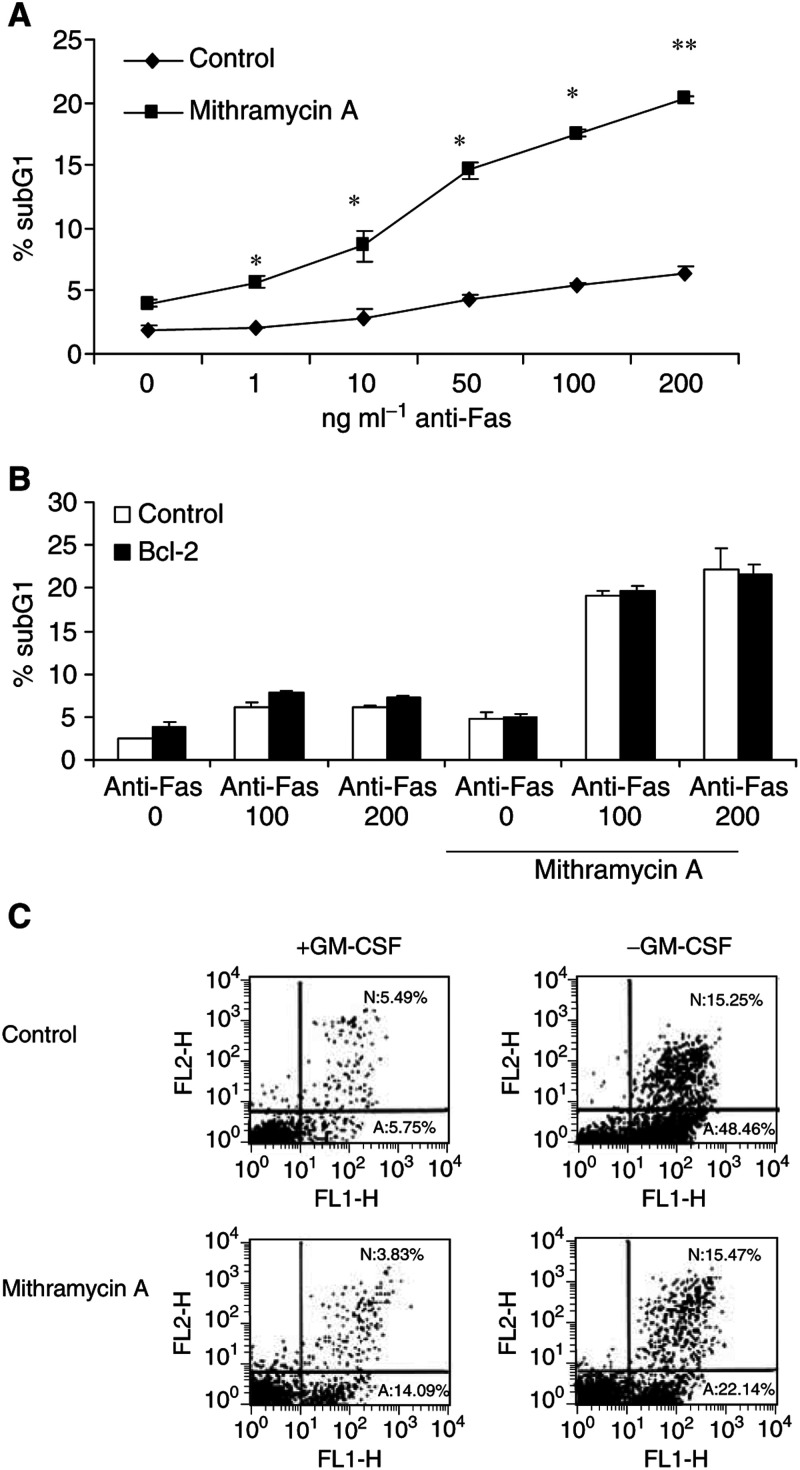
Sensitisation to Fas-induced, but not to GM-CSF withdrawal-induced apoptosis by mithramycin. (**A**) TF-1 cells were treated with increasing concentrations of anti-Fas antibodies in the presence or absence of 75 nM mithramycin. After 48 h of incubation, percentage of subG1 cells were determined by PI staining and analysed by flow cytometry. ^*^, *P*<0.05; ^**^, *P*<0.01 of three independent experiments. (**B**) Mock-transfected (control) TF-1 cells and cells stably transfected with GFP/bcl-2 (bcl-2) were treated with 100 or 200 ng ml^−1^ anti-Fas antibodies in the presence or absence or 75 nM mithramycin. After 24 h of incubation, percentage of subG1 cells were determined by PI staining and analysed by flow cytometry. Data are representative of three independent experiments performed in duplicate. (**C**) TF-1 cells were depleted of GM-CSF in the presence or absence of 75 nM mithramycin. After 48 h of incubation, cells were collected and stained with PI and annexin V. Necrosis (N) and apoptosis (A) were quantified by flow cytometry. Similar results were obtained from two more independent experiments.

Since both TNF and Fas receptors engage a common component to the apoptotic machinery (Chinnaiyan *et al*, 1996), we tested whether mithramycin would also have a synergistic effect on apoptosis in response to Fas ligation in TF-1 cells. TF-1 cells were treated with Fas agonistic antibodies in the presence or absence of mithramycin, and then assessed for DNA fragmentation. There was little effect on the viability of TF-1 cells after 48 h exposure to either agent alone; however, in the presence of both mithramycin and anti-Fas antibodies, TF-1 cells underwent significant cell death (Figure 5A

). Similar to TNF/mithramycin combination, induction of apoptosis by anti-Fas and mithramycin cotreatment was not blocked by bcl-2 overexpression (Figure 5B).

Next, to study whether the observed sensitisation was specific to death receptor signalling events, we tested the effect of mithramycin on apoptosis in response to growth factor depletion. Mithramycin appeared to delay the induction of apoptosis in TF-1 cells following GM-CSF removal (Figure 5C). GM-CSF depletion is reported to trigger apoptosis independent of death receptor signalling (Raff, 1998; Klampfer *et al*, 1999). Furthermore, we demonstrated in Figure 4B that overexpression of Bcl-2 prevented apoptosis after growth factor depletion. Altogether, these data would suggest that mithramycin-mediated sensitisation depends on the signalling pathway involved in the induction of apoptosis, and that the proapoptotic function of mithramycin involves death receptor-associated signalling events.

### Pretreatment is sufficient to significantly increase TNF-induced DNA fragmentation. Comparison with actinomycin D and aphidicolin

**Figure 6:**
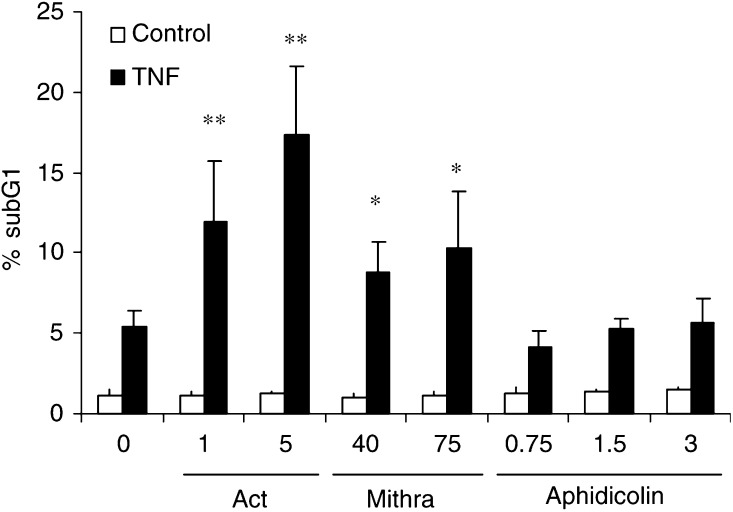
Enhancement of TNF-induced apoptosis by preincubation with mithramycin. Comparison to actinomycin D and aphidicolin. TF-1 cells were either left untreated or were pretreated with actinomycin D (1 and 5 *μ*g ml^−1^), mithramycin (40 and 75 nM) or aphidicolin (0.75, 1.5 and 3 *μ*M) for 4 h, followed by incubation with 20 ng ml^−1^ TNF for 24 h. Percentage of subG1 cells were determined by PI staining and analysed by flow cytometry. ^*^, *P*<0.05; ^**^, *P*<0.01 of three independent experiments compared to untreated cells.

Next, we examined whether pretreatment with mithramycin sensitises TF-1 cells to TNF-induced DNA fragmentation. As shown in Figure 6

, pretreatment with mithramycin for only 4 h significantly enhanced sensitivity to TNF-induced apoptosis.

Several studies indicate that transcriptional or translational inhibition enhanced the cytotoxic effect of death receptors in various types of cancer (Fulda *et al*, 2000; Wajant *et al*, 2000). Others studies demonstrate the involvement of DNA injury in potentiating TNF-induced apoptosis in cells treated with nontoxic concentrations of aphidicolin (Gera *et al*, 1993). Since mithramycin is reported to inhibit transcription and prevents DNA replication too, both effects of mithramycin could explain the sensitisation to TNF-induced apoptosis. To distinguish between these two effects, mithramycin-mediated sensitisation was compared to transcription and replication inhibitors, namely, actinomycin D and aphidicolin, respectively. In Figure 6, the percentage of cells with fragmented DNA in TF-1 cells pretreated with actinomycin D, mithramycin and aphidicolin and then incubated with TNF is represented. Whereas actinomycin D increased TNF-induced DNA fragmentation, the inhibitor of DNA replication aphidicolin had no effect on TNF-induced DNA fragmentation. These results suggest that the sensitising effects of mithramycin could be explained as a result of transcription inhibition. Mithramycin-mediated sensitisation to TNF-induced apoptosis may prevent the synthesis of short-lived inhibitors of apoptosis.

### Mithramycin-induced TNF sensitisation does not prevent NF-*κ*B activation

**Figure 7:**
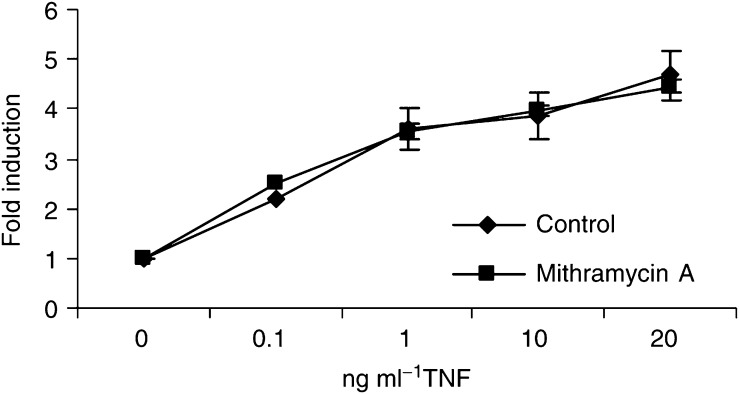
Mithramycin had no effect on NF-*κ*B-dependent gene expression in response to TNF. TF-1 cells stably expressing an NF-*κ*B-dependent hrGFP reporter gene were either untreated or treated with 75 nM mithramycin and stimulated for 10 h with a serial dilution of TNF. Cells were then assayed for hrGFP expression by flow cytometry. Data are representative of two independent experiments performed in duplicate.

In addition to the induction of the proapoptotic pathway, TNF activates the transcription factor NF-*κ*B, and this response potently suppresses the apoptotic potential of these stimuli *in vitro* (Beg and Baltimore, 1996; Ponton *et al*, 1996; Van Antwerp *et al*, 1996). To analyse whether mithramycin increased TNF-induced cell death by preventing TNF-induced NF-*κ*B activation, we studied the effect of mithramycin on the TNF-induced expression of an NF-*κ*B-dependent reporter gene in TF-1 cells. TF-1 cells stably expressing an NF-*κ*B-dependent hrGFP reporter gene were either untreated, or treated with 75 nM mithramycin and simultaneously stimulated for 10 h with a serial dilution of TNF. As shown in Figure 7

, no effect of mithramycin on NF-*κ*B-dependent gene expression was detected, indicating that mithramycin did not prevent NF-*κ*B activation following TNF stimulation.

### Decrease in cFLIP protein level after treatment with TNF and mithramycin

**Figure 8:**
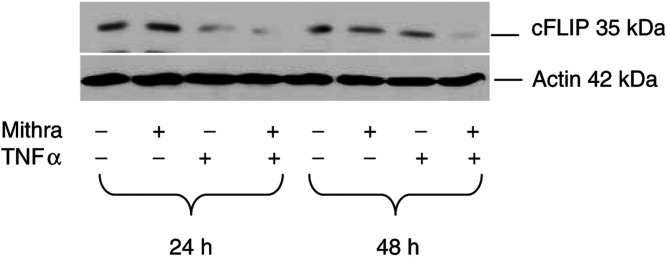
Decreased level of cFLIP protein in response to TNF and mithramycin. TF-1 cells were treated with 20 ng ml^−1^ TNF in the presence or absence of 75 nM mithramycin for 24 or 48 h. The expression levels of short-spliced cFLIP variant and actin were then determined by Western blot analysis.

Since TNF-induced apoptosis, but not TNF-mediated NF-*κ*B activation, required the presence of mithramycin, we hypothesised that the mithramycin-sensitive factor has to interfere with TNF signalling downstream of the bifurcation point of apoptosis and NF-*κ*B activation. A known receptor-proximal regulator of TNF- and Fas-induced apoptosis is the cellular FLICE inhibitory protein (cFLIP). We therefore investigated the expression levels of the short-spliced form of cFLIP in TF-1 cells. As shown in Figure 8

, the expression level of FLIP remained unaffected in mithramycin-treated cells. Treatment of the cells with TNF alone appeared to decrease the level of cFLIP protein at 24 h, however, expression levels recovered by 48 h. When cells were simultaneously treated with TNF and mithramycin, levels of cFLIP protein significantly decreased at 24 h, and no recovery in the protein expression was detected at 48 h.

## DISCUSSION

This current report demonstrates that a combination of TNF and mithramycin enhanced apoptosis of TF-1 cells within 24 h, relative to single treatments of TNF or mithramycin alone. Apoptosis induced by TNF and mithramycin treatment was effectively blocked by treatment with z-VAD-fmk peptide, an inhibitor of caspases, indicating that caspases played a critical role in the execution phase of apoptosis induced by TNF in the presence of mithramycin. Studies on the involvement of the mitochondria in the regulation of apoptosis revealed that TF-1 cells overexpressing the antiapoptotic protein Bcl-2 were not protected against apoptosis induced by TNF and mithramycin. These results further indicate that in the presence of mithramycin, TNF induced apoptosis via a caspase-signalling cascade that executed apoptosis independently of the proapoptotic machinery of the mitochondria. These results support the hypothesis that mithramycin targets an inhibitor of apoptosis involved in TNF-induced apoptosis, at the level of death receptor signalling.

Mithramycin also significantly increased apoptosis mediated by another member of the death receptor family, Fas (CD95/APO-1), and could not be suppressed by overexpression of Bcl-2, suggesting the existence of a common mithramycin-sensitive inhibitor of apoptosis. TNF- and Fas-induced signalling pathways leading to caspase activation and apoptosis converge at the level of FADD (Chinnaiyan *et al*, 1996). Fas directly binds to FADD, whereas p55 TNF-receptor initiates FADD clustering via the adaptator protein TRADD. This strongly suggests that the potentiating effect of mithramycin on Fas and TNF cytotoxicity is situated at the level of, or downstream of, FADD.

In contrast to the above findings, the current study also demonstrated that the presence of mithramycin is able to abrogate cell death induced by growth factor depletion. These observations are currently the focus of another study, however, it is in agreement of earlier observations that GM-CSF withdrawal in factor-dependent cell lines induces apoptosis in a manner independent of death receptor signalling (Raff 1998; Klampfer *et al*, 1999).

In the present study, we report that the sensitisation effect of mithramycin to TNF-induced apoptosis could be explained as a consequence of inhibition of mRNA synthesis. In addition, pretreatment of cells with mithramycin enhanced TNF-induced apoptosis, supporting that inhibition of short-lived repressors may account for the sensitisation effect of mithramycin.

We have found that in TF-1 cells, TNF activated NF-*κ*B in reporter gene assays, without concomitantly inducing cell death. Similarly, a combination of mithramycin and TNF was shown to activate NF-*κ*B in a fashion similar to TNF alone. These data argue for the existence of a mithramycin-sensitive inhibitor that blocked TNF-induced apoptosis but not TNF-mediated activation of NF-*κ*B. TNF-mediated NF-*κ*B activation and TNF-induced cell death bifurcate at TRADD. As in TF-1 cells, TNF-induced apoptosis, but not TNF-mediated NF-*κ*B activation, required the presence of mithramycin, and it is postulated that a mithramycin-sensitive regulator of apoptosis interferes with TNF signalling downstream of TRADD.

Altogether, FADD is the most likely target of the postulated mithramycin-sensitive factor, as (i) FADD is a common mediator of Fas and TNF receptor-induced apoptosis, (ii) in the TNF-receptor signalling complex, FADD is immediately downstream TRADD, a molecule that allows TNF-mediated NF-*κ*B activation in the presence of mithramycin. Cellular FLICE inhibitory proteins are capable of inhibiting apoptosis induced by several apoptosis-inducing receptors, through binding to FADD and thus preventing the recruitment of caspase-8 (Tschopp *et al*, 1998; Bin *et al*, 2002). Previous reports demonstrated that the transcription inhibitor actinomycin D sensitised cells to TNF-induced apoptosis through downregulation of cFLIP gene expression (Fulda *et al*, 2000; Kim *et al*, 2000). Similar to actinomycin D, mithramycin-mediated sensitisation could be explained by the transcription inhibition of apoptosis inhibitors. Interestingly, mithramycin alone did not affect the protein level of cFLIP, but strongly downregulated the expression level of cFLIP in combination with TNF. This would suggest that, in contrast to the nonspecific transcription inhibitor actinomycin D, mithramycin may prevent an inducible pathway that triggers transcription of antiapoptotic genes, such as cFLIP. Thus, given its selective and low cytotoxic effects, mithramycin may offer a better alternative to metabolic inhibitors as an anticancer agent.

The ability of mithramycin to enhance the direct cytotoxic effects of TNF (and TNF family members) has wide ranging therapeutic implications. Since the mithramycin/TNF combination is capable of inducing apoptosis in TF-1 cells independently of the mitochondrial pathway, combining TNF treatment with mithramycin could have potential for elimination of tumour cells that express large amounts of Bcl-2. In addition, apoptosis induced by TNF in the presence of mithramycin was enhanced in three cell lines with different p53 status. As loss of p53 function contributes to resistance of some tumour cells to TNF-induced cytotoxicity (Cai *et al*, 1997), the present study suggests that combination of TNF with mithramycin may be a potential strategy to sensitise mutant p53 TNF-resistant tumour cells.

Recent data indicate that induction of tumour cell death by stimulation of death receptors such as Fas constitutes a more prominent mechanism in the defence against tumours than has been thought previously (French and Tschopp, 2003). As blockade of death receptor-mediated apoptosis has been implicated in treatment resistance *in vivo*, strategies to increase death receptor-mediated cell death, for example by mithramycin, may prove to be a useful complementary tool for treatment of cancer. Alternatively, given the similarity in inducing apoptosis between death receptors, mithramycin may be expected to enhance tumour necrosis factor-related apoptosis-inducing ligand (TRAIL)-induced cytotoxicity, thus representing a novel alternative in cancer therapy. Indeed, TRAIL or Apo-2L, a member of the TNF family, induces apoptosis preferentially in tumour cells also through binding to its cognate death receptors and recruitment of FADD (Walczak and Krammer, 2000). Unlike Fas ligand and TNF, TRAIL appears to have limited toxicity for mice and monkeys (Walczak *et al*, 1999), suggesting its potential value for cancer therapy.

Since mithramycin has been used for many years in the clinic to treat several types of cancer, the sensitising effect of mithramycin is not only intriguing but merits further study to realize the full potential of mithramycin as an adjunctive therapy in death receptor-mediated apoptosis.
